# The Nature and Treatment of Cancer

**Published:** 1846-10

**Authors:** 


BRITISH AND FOREIGN

MEDICAL REVIEW,

FOR OCTOBER, 1846.

### PART FIRST.

### Analytical and Critical Reviews.

### Art. I.

*The Nature and Treatment of Cancer.* By W. H. Walshe, M.D., Professor of Pathological Anatomy in University College, Physician to University College Hospital, and to the Hospital for Consumption and Diseases of the Chest.—*London,* 1845. 8vo, pp. 590.

We proceed with our analysis of the second division of this valuable work, in which the disease is described as it affects various organs, tissues, and parts.

CANCER OF THE DIGESTIVE ORGANS AND APPENDAGES.

1. *Lips.* Labial cancer, without being very common, is of tolerable frequency. There is some variety in its mode of origin and progress. It may commence by infiltration of scirrhus in a limited portion of the lip, underneath the skin, at its junction with the mucous membrane. After a time one of these tissues gives way, leaving a fissure which discharges a thin acrid fluid that dries into a scab. When this falls, or is torn off, it is reproduced, until, as the ulceration advances, no more regular scabs are produced, but fungous granulations spring up from the scirrhous basis. In some cases, the first symptom is said to be a fissure, which resists all treatment. It has been known to commence as a cutaneous warty excrescence, or, as described by Dr. Warren, as a pustule; and it is most certain that venereal ulcerations of this part sometimes become cancerous, though the frequency of such an event has been much exaggerated. But, however commencing, as the disease advances, the skin, mucous membranes, and labial glands form a prominent mass, which, combined with the fungous productions from the sore, produce an abundant slaver that constantly escapes over the lip, and irritates or excoriates the neighbouring skin. The ulceration may spread over the entire cheek, and even part of the external ear. It may affect the maxilla, and has even been known to reach the sternum. The salivary and lymphatic glands become enlarged and painful, and, especially the latter, may partake of the diseased action. General contamination of the system is comparatively rare. Scirrhus is the usual form of cancer in this part; encephaloid has rarely, if ever, been observed. It is excessively rare in females; and is almost invariably seated in the lower lip. The only affection with which it is likely to be confounded is *venereal ulceration with indurated base;* in many cases the history is the only diagnostic guide.

When the ulceration is superficial, it may sometimes be successfully treated by caustics, the acid nitrate of mercury and the chloride of zinc being the best. It is generally supposed that excision is more successful here than in other parts; but there has been much exaggeration on this point.

2. *Gums.* The term *epulis* is applied to all solid growths from the gingival membrane of either jaw. Cancerous epulis is generally of the scirrhous species. It is more common in the lower than the upper jaw, and usually appears at the reflexion of the membrane on the alveolus, towards the last incisor or bicuspid teeth. It commences by infiltration; but a tuberculated nodule, sometimes with very deep anfractuosities, soon results. It generally varies in size between that of a pea and a nut, but has been said to attain the bulk of a walnut. Ulceration eventually takes place, the sore fungates and sometimes bleeds, and the discharge is peculiarly fetid. The disease destroys the soft parts, and may, but rarely, affect the bone. It is most common in advanced age and in males. There is usually lancinating pain. It is distinguished from the flabby, fungous granulations which sometimes form in the gums, near carious teeth, by its density, its tuberculated shape, and pale colour; and from erectile epulis, by the pulsating characters of the latter. From simple epulis it can scarcely be known, excepting from the result of operation.

3. *Tongue.* Lingual cancer is tolerably frequent. Out of 8289 fatal cases of cancer, reported in the Paris registers, in 36 the main disease was seated in the tongue. Scirrhus is the usual form; but the fungous excrescences sometimes exhibit the encephaloid character. It may commence as a small, somewhat knotty, and irregular tumour, which is generally seated in the anterior part of the organ, midway between the raphe and the edge, and rarely extends beyond the middle line; or the first appearance is a small excrescence, which in some instances is said to become pedunculated. In a few rare cases simple ulcers become cancerous; and still more rarely the morbid matter is deposited in erectile tumours. In either case the surface ulcerates, and the glands become affected in the usual way. Distant organs are not commonly implicated. Acute pain is not a constant symptom; but there is generally an aching sensation, with occasional sharp pangs, darting towards the ear. One of the most distressing concomitants is the necessity for incessant sputation. There are, of course, pain and difficulty in speaking, mastication, and deglutition. These increase with the progress of the disease, until the performance of these functions becomes impossible. The cachexia is frequently intensely marked, and the sufferer is commonly cut off by its progress. According to Mr. Travers, strong and healthy males, æt. 40 and upwards, are the most usual subjects; but the disease is not unfrequently observed in females, and much younger persons.

Lingual cancer may be confounded with *simple induration* of one side of the base of the tongue. The situation and absence of constitutional symptoms will distinguish between the two. Mr. Travers describes a *globular tumour* seated deeply in the organ, which is recognized by its uniform and unyielding surface, and by its disappearance under the use of alkalies and tonics. *Simple ulceration,* produced by mechanical irritation, is usually known by its rapid cure after removal of the cause. *Syphilitic ulcers* are less easily distinguished; they are generally larger, and have less marked and less circumscribed surrounding hardness. The discharge is also less abundant, and they are deficient in the firm everted edge and sprouting fungi. In many cases the history is our only guide. The *fissured* or *dyspeptic ulcer,* which often originates in psoriasis, may bear a close resemblance to cancer, but it has not the hard basis, is often in the middle line, and the rest of the tongue is chapped and irregular. Dr. Warren describes an *enlargement of the mucous glands,* occurring on the side of the tongue, with a red fungous appearance, but differing from cancer in being sensitive, not painful, and unattended by real ulceration or thickening of the organ. *Hypertrophy of the mucous membrane* sometimes gives rise to irregular fissured elevations on the surface. *Hypertrophy of the tongue generally,* a most rare malady, can scarcely be confounded with cancer. *Erectile tumours* are known by their pulsation; but it must be remembered that they may become the seat of cancerous deposits.

In regard to treatment little can be said. Ablation may be practised to avert impending death; but as a curative measure there is no evidence in its favour.

We pass over the description of cancer of the hard and soft palate, the tonsils, and salivary glands; but must say a few words on *pharyngeal carcinoma.* This is rare. Four out of the 8298 cases in the Paris registers were of this kind. The infiltrated form of scirrhus is the most usual species. The disease first presents itself as a hard, imperfectly circumscribed mass, situated in the submucous tissue, and not painful on pressure. Eventually it ulcerates, and passes through the ordinary stages. The symptoms are dysphagia, slight at first, but gradually increasing until deglutition becomes impossible; painful regurgitation of the discharges, and, if the larynx become involved, dyspnea. Hemorrhage is sometimes the first symptom. The patient commonly dies of inanition; and the treatment can be merely palliative. The diagnosis is not always easy. *Simple induration,* with thickening of the submucous tissue, will occasion the same dysphagia, and can only be distinguished by the result of treatment and by the progress of the disease. *Sacculation of the walls of the pharynx,* which also produces some of the symptoms, may be known by the facility with which instruments can generally be passed. *Venereal ulcerations* will be recognized by the history; *polypi* by their form, which is never assumed by cancer in this place; and *enlargement of the cervical vertebræ* by manual examination.

*Œsophagus.* The registers above noticed give 13 deaths from cancer of this organ. It is most frequently seated in the upper end of the canal behind the larynx, most rarely at its middle part, but it may occupy the whole extent of the tube. Infiltrated scirrhus deposited in a stratiform manner is the usual condition, and it generally commences in the submucous tissue. Distinct scirrhous tumours are comparatively very uncommon; and tuberiform encephaloid still more so. Distant organs are rarely implicated. The *nature* of the symptoms is the same as when the disease is situated in the pharynx, but as the stricture is lower down, the first stages of deglutition are performed naturally. Subsequently, if the upper end is affected, the greater proportion of each mouthful of food at once regurgitates; if a lower part, three or four mouthfuls pass easily, and the accumulated matter is then gently expelled by the action of the muscular fibres. These occurrences are at first only occasional, but eventually the dysphagia becomes permanent. It is necessary, however, to bear in mind that the disease may be intermittent throughout. The œsophagus sometimes forms a large pouch above the stricture, in which the ingesta accumulate; and here, according to Mr. Purton, they undergo a species of chymification. The progress of the disease is accompanied with pain, and obstinate hiccup is not uncommon. Spasmodic dysphagia is a frequent complication, and when some of the food passes into the stomach, it is often rejected by vomiting. The cachexia is commonly marked, and the patient dies of inanition. There is also danger of severe hemorrhage. Perforation may also take place, with effusion into the mediastinum, larynx, trachea, bronchi, pleura, or pulmonary tissue. The diseases simulating carcinoma of this tube are *spasmodic stricture* and *spasm of the diaphragm*, to be recognized by the history, and the result of treatment; *paralysis* of the muscular coat, known by the easy passage of instruments, and the fact that large masses are more easily swallowed than small ones; *dilatation* and *sacculation* of the tube, also to be diagnosed by the probang; *hypertrophy* and *induration* caused by inflammation, this is generally the result of the ingestion of poisons, or the lodgment of some mechanical irritant, and the history is therefore our guide. Enlarged glands, abscesses, aneurisms, &c., may also cause great obstruction, but a careful examination will prevent error. The treatment can be merely palliative; but as some organic strictures have been cured by persevering dilatation, it will be well to give this plan a trial, and probably Dr. James Arnott's system of fluid pressure would be the best method. Leeching the throat often gives great relief, and benefit is sometimes derived from the application of the lunar caustic, to alter the condition of the sore, when ulceration has taken place. The œsophagus tube may be necessary, and if the disease is situated at the upper part, life might be prolonged by making an opening below the obstruction.

*Stomach.* The mortality from cancer of the stomach is very considerable. In this respect it yields to no organ but the uterus. Of the 8289 deaths in Paris, 2303 are referred to the stomach. In 67 cases, MM. Herrick and Popp found this organ diseased in 19. Cancer of the stomach may exist alone, but is usually associated with similar affections of other organs, more especially of the liver. It is almost invariably primary. All the varieties of the three species of carcinoma occur in this organ; it is the special site of colloid cancer; and the milt-like variety of encephaloid is more common here than elsewhere. Infiltration is essentially the mode of deposition in this place, and, indeed, throughout the alimentary canal; and the seat of the deposit is the submucous cellular tissue, though the mucous membrane, particularly when hypertrophied, may become a nidus of formation. The pylorus is the part most commonly affected; next the cardiac orifice; then the greater, and, lastly, the lesser curvature. Dr. Walshe has not met with an instance in which the disease was limited to the fundus—a fact of importance as bearing upon the supposed origin of the disease from the ingestion of irritants. Three fourths, or even more, of the organ may be disorganized; and this is particularly the case when colloid is the species.

The mucous membrane long resists the disease, and its chief tendency is to become irregularly hypertrophous, giving rise to the apparent formations of vegetations, &c. The cellular structure undergoes very marked thickening, and this occurring between the muscular fibres produces the striated appearance so commonly observed. The muscular coat of the sound parts of the organ is often enormously hypertrophied. The peritoneal coat is seldom affected, excepting in cases of colloid.

The size of the organ varies extremely. When the pylorus is affected it is often greatly enlarged; when the cardiac orifice, it is contracted; when the body of the organ is alone diseased, the general bulk commonly remains unchanged. It is a curious and unexplained fact, that, where the pyloric orifice undergoes dilatation, the walls of the stomach become hypertrophous.

The progress of the disease presents nothing peculiar. Ulceration is slow to commence; but when once begun, its ravages are extensive. In most cases, adhesions, especially to the liver and pancreas, take place before the peritoneum gives way.

The disease is more common in males than in females, and between the ages of 35 and 60. It is often hereditary, and has apparently, in many cases, been induced by mental distress.

Cancer of the stomach, in the early stages especially, may be confounded with other affections; the most practically important of which are *gastrodynia* and *chronic gastritis*. We subjoin the following sketch of the chief points of distinction:

| *Gastrodynia.* | *Chronic Gastritis.* | *Gastric Cancer (early period.)* |
|---|---|---|
| Tongue variable; but often pale, and pitted at the edges. | Tongue dry, red, contracted, smooth, shining, or saburral. | Tongue pale or natural. |
| Eructation frequent of air, without disagreeable smell. | Eructation not a prominent symptom. | Eructation of air more or less fetid, sometimes horribly so, a prominent symptom. |
| Appetite depraved, irregular, capricious. | | Appetite diminished, or even totally suppressed. |
| Sensations, sometimes of heat, sometimes of cold in stomach; thirst not common. | Sensation of heat in stomach; thirst. | These symptoms not observed. |
| Solids more easily digested than liquids. | | Liquids more easily digested than solids. |
| Digestion completed, though with much labour and suffering. | Digestion imperfectly completed. | Digestion not properly effected. |
| Pain variable, occurs in irregular paroxysms; is often relieved by ingestion of food or pressure. | Epigastric pain not very severe, and scarcely ever felt when the stomach is empty; increased by pressure. | Epigastric pain may be agonizing; the lancinating character sometimes marked; often increased by pressure. |
| Epigastric pulsation not uncommon. | Is not observed. | Is not observed. |
| Never runs a completely latent course. | Never completely latent. | May for a variable time be completely latent. |

| *Gastrodynia.* | *Chronic Gastritis.* | *Gastric Cancer (early period.)* |
|---|---|---|
| Chronic vomiting is most frequent in females, and is almost confined to persons affected with hysteria. | Vomiting of sudden and severe character sometimes the very first symptom; occurs irregularly before or after eating. | Vomiting of sudden and severe character is never the first symptom; it occurs generally early in the morning, subsequently at variable periods after eating, or at periodical intervals. |
| Vomiting of coffee-ground-looking matter does not occur, unless from accidental and rare hæmatemesis. | Coffee-ground-looking matter sometimes vomited; but this is rare and exceptional. | The matters vomited are at first glairy, then half-digested food, then coffee-ground or soot-like. |
| Bowels generally constipated, but not obstinately so. | Irritation, colic, and diarrhea frequent, from extension of inflammation to intestine. | Bowels habitually and obstinately constipated; occasional severe diarrhea. |
| Febrile action accidental and rare. | Evening fever not uncommon. | Fever absent. |
| In females the chlorotic tint is often present. | Violet discoloration of the lips, conjunctivæ, face, &c., often present. | Straw-coloured tinge of skin may be obvious. |
| Often accompanied with various nervous or hysterical symptoms. | Not so attended. | Not so attended. |
| Hypochondria occasionally present. | Hypochondria not caused by chronic gastritis. | Hypochondria not an effect of gastric cancer. (?) |
| Is more frequent than the other two. | Is rarer even than cancer. | Is much rarer than gastrodynia. |
| Is more common in women than men. | Is probably equally frequent in both sexes. | Occurs more frequently in men than women. |
| May exist in very young persons (e. g. æt. 15). | Occurs at all ages. | Is excessively rare before æt. 30. |
| Is often hereditary. | Is not hereditary. | Occasionally runs in families. |
| Is rarely referrible to any distinct local exciting cause. | Is often referrible to some distinct local exciting cause. | Is rarely, if ever, referrible to local agencies. |
| Is relieved or cured by stimulant, tonic, and anodyne treatment. | Is relieved or cured by antiphlogistic treatment. | Is not cured, but is relieved by special treatment. |

As the disease advances the diagnosis becomes less obscure, but cases do now and then occur which present all the ordinary combinations of symptoms, and yet are not cancerous; so that the detection of *tumour* is the only absolutely certain sign. This is most easily discovered when seated in the pylorus or great curvature: it is much more difficult to detect when occupying the lesser curvature or the cardiac orifice. It must be remembered that the situation of the tumour changes continually; and it is of the last importance to bear in mind that the whole course of the disease may be of an intermittent character.

In regard to treatment but little can be said. Conium is the only supposed specific which is applicable. Dr. Walshe has derived benefit from a combination of trisnitrate of bismuth and extracts of hop, stramonium, and conium, in pill; and he believes that the treatment generally should be such as is applied to nervous, rather than inflammatory affections. Leeches and blisters may be used to relieve occasional local irritation. Opium is contraindicated by the constipation; but the Indian hemp may be tried. A drop or two of oil of cajeput on sugar is the safest carminative. Cold applications or a blister sometimes also relieve the flatulence, and a dose of morphia has been found useful in the same way. But most is to be done by regulating the diet, observing what agrees with the patient, making the *quantity* taken at each meal *small*, securing perfect regularity in the hours, and complete mastication of each morsel. Sickness may be relieved by effervescing draughts, prussic acid, blisters, rough ice allowed to melt in the mouth, the application of ice in bladders to the epigastrium, or an occasional dose of creosote. The bowels are best kept open by enemata: drastic purges are quite inadmissible.

M. Recamier narrates a case of pyloric tumour, "possibly cancerous," which was reduced in bulk by pressure, applied by means of a folded napkin secured by a bandage round the body. It may be well to try some such plan as this.

*Intestines.* The intestinal canal is among the more frequent seats of cancer. The Paris registers give 378 out of the 8289 deaths; in 221 of these the disease occupied the rectum, showing the much greater liability of the great than of the small intestines to affections of this nature.

Cancer of the small intestine is almost invariably primary: it is most commonly found in the duodenum and upper part of the jejunum, and is so rare in the ileum that its existence there has been altogether questioned. The symptoms are those of chronic intestinal disease complicated with evidences of increasing obstruction, passing eventually into a state of strangulation, but the actual diagnosis must depend upon the discovery of a tumour. The treatment is similar to that of cancer of the stomach.

Cancer of the rectum and anus are so well known, that we need not delay upon them. Dr. Walshe speaks in anything but favorable terms of the operation for extirpation, and would limit its performance to those cases in which, from the completeness of the obstruction, speedy death appears inevitable, and when the use of bougies has failed to give even temporary relief. But even under these circumstances the surgeon has his option of making an artificial anus, either in the groin, in which case the peritoneum must be opened, or (according to Callisen's method, modified by Amussat) in the lumbar region, without injuring that membrane. For our own parts, we are inclined to give our decided preference to the last-mentioned operation.

Primary cancer is sometimes, but very rarely, found in the *mesenteric glands.* Dr. Carswell has shown that the disease may be seated on the free surface of the *peritoneum;* and the omenta, especially the *omentum majus,* is not infrequently affected, secondarily, with colloid.

The *post-peritoneal cellular tissue* is by no means an uncommon locality of cancer, and the tumours, which are usually composed of scirrhus and encephaloid in variable proportions, often rapidly acquire an enormous size without giving rise to any marked symptoms.

Cancer of the *spleen* is of so little practical importance that it may be passed by unnoticed.

The *pancreas* is among the rare sites of cancerous formation. It has been denied that the disease is ever of primary occurrence in this organ, but such an opinion is contradicted by well ascertained facts, and, indeed, it would appear that its secondary affection is the least common. Both scirrhus and encephaloid are found here; the former is usually infiltrated, the latter is developed in the form of tumour. Generally a part only of the gland is affected, the head being perhaps the most frequent seat. The symptoms are obscure, and of course vary according to the influence which the diseased mass exerts over adjacent organs. There is usually pain; often vomiting; in many instances, but less invariably than has been thought, an abundant watery discharge from the mouth, which discharge is supposed, but not proved, to proceed directly from the pancreas; and, when the tumour has ulcerated, discharge of blood from the stomach and by stool. It should be observed, however, that the absence of such bloody discharge is no decided evidence that softening has not taken place.

*Liver.* The liver stands pre-eminent among all the organs of the body for the frequency of its cancerous affections—even the primary form is extremely common. And of all its organic diseases cancer is the most frequent; but, when occurring primarily, it is slow to contaminate distant parts. Encephaloid is found here in the pure cerebriform state, and in the solanoid and hematoid varieties; scirrhus chiefly in the chondroid and napiform varieties. Dr. Walshe has never seen colloid in this organ. The most common combination is scirrho-encephaloid.

When primary, cancers in the liver are usually limited in number; when secondary, they are often exceedingly numerous, and this is peculiarly the case when the stomach is the part first affected. The lobules appear to be the seat of deposition. Dr. Walshe has invariably found, on examining a section of the liver, adjoining cancerous nodules, with a common lens, that the surface is sparsely studded with minute milky, though somewhat transparent, spots, looking as if the discoloration, which softens off at the edges, were produced by the point of a brush. The lobules seem *infiltrated* with the milky-looking material, which is most abundant towards their centre. Their outline and size exhibit no obvious change.

Our author has never seen the general bulk of the liver reduced notably below the natural standard, but in some instances absorption of its substance proceeds so uniformly with the morbid deposition, that no enlargement occurs. In other cases the dimensions of the organ are greatly increased. The existence of the one or other of these states makes a vast difference as regards the facility of diagnosis.

The disease is more frequent in males than females, and is most common between the ages of 50 and 70. Many of the symptoms are the same as in cancer of the stomach: those which specially point to the liver are tumour in the hypochondria, jaundice, and ascites. When the tumour can be felt it is nodular on the surface: this distinguishes it from fatty enlargement. Jaundice, without ascites, occurs in somewhat less than half the cases. Ascites, without jaundice, is much less frequent. The two combined occur in a fourth or fifth of all cases; in about a third neither is present.

The treatment should be generally conducted as that of gastric cancer. Dr. Walshe thinks he has seen the progress of the disease stayed by liberal inunction of the iodide of lead ointment over the hepatic region, and the internal administration of liquor potassæ in infusion of taraxacum.

### CANCER OF THE RESPIRATORY ORGANS AND APPENDAGES.

Passing by the cancerous affections of the *nares*, *frontal* and *sphenoid sinuses*, *epiglottis*, *larynx*, *trachea*, *bronchi*, and *bronchial glands*, all of which are noticed, more or less fully, by our author, we arrive at the *lungs*; and here we must delay for a little space, remarking *in limine* that the reader will find in this section the best and most comprehensive account of the subject that we have anywhere met with. In 1091 post-mortem examinations of persons dying of all diseases, MM. Herrick and Popp found the lungs cancerous 6 times. In 67 cases of cancer these organs were affected 6 times. Of the 8289 cases included in the Paris registers, the fatal event is ascribed to the lungs in 8. The disease is most common as a secondary affection; when primary it shows little tendency to contaminate the system. Encephaloid is the species most frequently met with. The right lung is the most common seat of primary cancer ; in the secondary affection both are usually implicated.

Secondary cancer generally exists in the nodular or tuberous form, more rarely as stratiform and cupulated sub-pleural patches; yet more rarely as a large irregular mass; and, least commonly of all, infiltrated through the pulmonary tissue. Primary cancer is found as an irregular mass in the majority of cases. It frequently occurs in the infiltrated form; very rarely in the nodular shape. It appears as the sub-pleural stratiform patch, or the supra-pleural nodule; is often both in the infiltrated and irregularly tuberous form in the same lung; and may exhibit itself as a series of fringed processes hanging from the free border of the organ.

All parts of the lung are liable to the disease, but the different forms affect a preference for particular localities. Thus, the disseminated nodular mass occurs scattered through the lung generally, but has a special tendency to accumulate near the pleura. The irregular tuberous mass is commonly found in the middle lobe of the right lung, whence infiltration extends in the majority of cases, though it may proceed from the apex towards the base, or *vice versâ*. It is probable that the intervascular interstices of the lobules are the main seat of formation. The secondary nodule doubtless originates in stagnation occurring in the ultimate radicles of the pulmonary vein. The progress of the disease is the same here as in other parts. The nodular form rarely produces any effect on the pulmonary tissue, except detrusion, with or without condensation, or atrophous rarefaction. A large tumour, of course, gives rise to condensation. Pneumonic consolidation is rare. Gangrene and pulmonary apoplexy have been observed. The ultimate branches of the pulmonary artery and vein are sometimes obliterated through extreme compression: in a case recorded by Heyfelder these vessels were converted into ligamentous cords from the heart onwards. It remains to be ascertained, whether, in such instances, a new circulating system is established round the affected parts, as Van der Kolk and Guillot have shown to arise in tuberculization. The trunk and larger branches of the pulmonary vein not unfrequently contain masses of the morbid deposit, and the same may occur in the superior cava. The bronchi undergo compression, or sometimes obliteration ; or they may be partially destroyed by the extension of ulceration; or they are perforated by a portion of the morbid formation, which then vegetates in their interior. When a chief bronchus passes through the growth, and is there compressed, dilatation may take place above the obstruction. Little notice has been taken of the condition of the mucous membrane. The pleura is invariably in an unhealthy state. It is a fact of great practical significance, that in no case have phthisical tubercles, in any form or stage, been observed, and that ulceration of the small intestines has not been noticed.

The growth of cancer of the lung is not limited to any particular period of life, but the male sex appears to be more prone to the disease than the female. In 10 cases the mean duration of the disease (calculated from the first appearance of symptoms) was 13·2 months; the greatest 27· months; and the least 3·5 months.

In most cases the disease, when of secondary type, runs an entirely latent course, giving rise to no symptoms, physical or functional; but this is far from being the state of matters in the primary affection. Here pain is almost invariably present; there is usually dyspnœa, and always cough, which in some few instances is dry, but generally accompanied by expectoration. The matter thus brought up varies in quality; it may be simply mucous or catarrhal, or purulent or bloody. In the latter case the blood seems mixed thoroughly with serosity, mucus or muco-pus, and the sputa, thus composed, are opaque and commonly free from viscidity, though they may be somewhat glairy, and in colour may be pink, or resemble red or black currant jelly. Actual hemoptysis is of frequent occurrence. It existed in 10 out of 19 cases analysed by Dr. Walshe, and of the remaining 9, in 4 there was bloody expectoration. This is a very high proportion, 72 per cent., while M. Louis gives 66 per cent. as the ratio in the consumptive patients observed by him. The decumbency is generally on the back, with slight inclination towards the affected side; in some cases there is orthopnœa towards the close of life. We have also as important symptoms the effects of *centripetal* and *centrifugal pressure;* the former exhibited in dysphagia, from pressure on the œsophagus; distension of the superficial veins, general tumidness of the face and neck, prominence and staring expression of the eyeballs, lividity of the surface and œdema, from compression of the deep venous trunks; and stridulous respiration, weakness of voice, and labour in speaking, from pressure on the trachea. The latter is shown by expansion of the walls of the chest, but this only occurs when the disease is of the tuberous form.

*Physical signs.* These are well marked and most satisfactory, as will be seen from the subjoined tabular view:

| (A) Simple cancerous infiltration, or infiltration associated with limited tuberous formation, the latter especially seated towards the root of the lung. | (B) Cancer of the lung mainly tuberous, but (it may be) associated with infiltration to a limited extent. |
|---|---|
| *Inspection.* | |
| Retraction or depression of the affected side, with more or less marked deepening of the intercostal spaces; diminished motions of expansion and elevation; diminished costal motions. | Diseased side expanded generally, but not with perfect uniformity, nor bulged inferiorly; intercostal spaces widened, flat, or even slightly fuller than natural; motions, both general and costal, completely abolished; fluctuation imperceptible in intercostal spaces. |
| *Application of the Hand.* | |
| Vocal and tussive fremitus diminished in intensity; impulse of heart distinctly perceptible over the anterior surface of implicated side in some rare cases. | Surface unnaturally smooth and even; vocal and tussive fremitus completely deficient; neither simple nor peripheric fluctuation; double pulsation sometimes transmitted from the heart. |

### *Mensuration.*

Semi-circular measurement natural or diminished; deficient increase of width during inspiration.

Semi-circular measurement of the side natural or increased; width of side unaltered by inspiration; antero-posterior diameter increased; vertical measurement increased in cases of very extensive formation; distance between the nipple and the median line greater than on the opposite side.

### *Situation of surrounding Parts.*

Mediastinum may be detruded towards the opposite side; the heart may be similarly displaced laterally or downwards; the diaphragm and infra-jacent viscera are not depressed.

Heart and mediastinum detruded laterally; corresponding division of the diaphragm with the infra-jacent viscera more or less depressed.

### *Percussion.*

Sound intensely dull and of short duration; resistance of walls strongly marked; special character of sound tubular in some cases about the lower border of the infra-clavicular region; the dullness of sound sometimes extends beyond the middle line.

Sound completely and extensively dull and of short duration; resistance of walls extreme; limits of the dull sound not altered by changing the position of the patient.

[Before softening of the cancerous matter.]

### *Auscultation.*

Strongly marked, diffused blowing respiration, or, with the progress of the disease (in consequence of accidental obstruction or obliteration of a chief bronchus), respiration weak or almost suppressed,—retaining, as long as it continues audible, the bronchial or blowing special character, bronchophony; bronchial cough; heart's sounds audible with undue intensity over the affected part.

Diffused or tubular blowing respiration, intensely developed in some cases; rhonchi either absent, or those of co-existing bronchitis; bronchophony; bronchial cough; heart's sounds audible with undue clearness; double blowing murmur in very rare cases accompanying the cardiac pulsation above mentioned.

[After softening and elimination of cancerous matter.]

*Percussion.* Sound may become somewhat clearer; acquiring at the same time more distinctly the tubular, almost amphoric, special character; the resistance of the walls may proportionally diminish.

*Auscultation.* Cavernous respiration; mucous, cavernulous, or cavernous rhonchus.

The unaffected lung is the seat of exaggerated respiration, and the percussion sound of the corresponding side may be fuller and clearer than natural.

We have been thus full in noticing the physical signs, because it is clearly by a correct appreciation of these that the physician will avoid errors in diagnosis: the original work contains many precise rules for his guidance, but we have not space to enter upon them.

The disease is necessarily fatal, and but little can be effected by treatment. Dr. Walshe has found the intense dyspnœa more relieved by large blisters, frequently repeated, than by any other measure.

We must refer to the original for an account of the little that is known regarding cancer of the *organs of circulation.*

### CANCER OF THE KIDNEY.

It is only of late years that the subject has attracted much attention, but our author has succeeded in collecting forty examples as the basis of his description. When cancer affects the kidney the disease is rarely limited to that organ, and in the great majority of cases its implication is secondary. Primary cancer is, however, less uncommon than is generally fancied. The associated organs vary extremely, but

M. Rayer has shown that the liver and right kidney, and the adjacent parts of the stomach or descending colon, and the left kidney, are often affected together. It is less common than might be expected to see the bladder and kidney simultaneously diseased. Encephaloid is by far the most common species; scirrhus is decidedly rare, and colloid has not been detected in this locality. When the disease is primary it is disposed to pass through its stages rather rapidly; its advance is slow when secondary. The destruction of the renal substance and softening of the cancer forms cavities, filled more or less with variously altered blood and detritus. These cavities may remain shut sacs, or communicate with and pour their contents into the calices or ureter, or, in rare cases, into the extra-renal cellular tissue, the peritoneal cavity, or the interior of some portion of the intestine.

Encephaloid occurs in the kidney in the infiltrated and tuberous forms; the former more especially when the disease is primary, the latter when secondary. Both originate in the cortical substance. In some cases the disease affects the various parts of the organ pretty equally; in others the morbid matter accumulates, especially at the convex surface of the kidney, or in its upper half, while the lower part remains perfectly untainted. As the disease advances the organ loses its natural shape: Dr. Walshe has seen it large enough to fill three fourths of the abdomen. The lymphatic glands and vessels are frequently implicated, and, by pressing on the duct, may give rise to great danger.

In 35 cases, the disease affected both organs 16 times; the right alone, 13 times; and the left, 6. It is most common in males, and in persons of advanced years, but may occur at any period of life.

In a few extremely rare instances the disease has remained latent until the patient's death; in others, pain of variable character has been the sole symptom throughout. Generally, pain is the only evidence of the malady until it has advanced so far that the tumour can be felt externally. The *direct* diagnosis depends upon the existence of this and another symptom, hematuria. The *tumour*, seated in the lumbar region, varies in size; it may scarcely protrude beyond the false ribs, or may reach the crest of the ilium; it may extend laterally beyond the umbilicus, and may obviously push forward the abdominal walls. In some cases it is distinctly visible and palpable in the lumbar region; in others there is no posterior intumescence. It is irregularly circumscribed, unevenly tuberous on the surface, unequally prominent, and unequally resistant in its different parts, variously elastic, but never really fluctuating. Whether the seat of spontaneous pain or not, it is almost invariably tender under pressure. Its dimensions are best ascertained posteriorly by the dull sound under percussion, which extends laterally to the flank; anteriorly, the amphoric note of the intestine is often producible over a limited portion of the dull sounding surface, from folds of intestine lying in front of the tumour—this is of importance in the diagnosis. The growth has usually been rapid.

In the early stages, the urine generally continues unaltered; but when the disease is advanced changes in that fluid are constant, and these consist essentially of the presence of blood or certain of its elements, of pus or encephaloid matter. *Hematuria* denotes either the breaking up of the tumour, or the coexistence of cancer in the calices or pelvis. It does not necessarily remain a continuous symptom, and the quantity of blood lost varies greatly. *Albuminuria* has been observed, but it does not persist for more than a few days after the cessation of hemorrhage. The discharge of *pus* is seldom very great; but the microscope would probably often detect the presence of encephaloid matter.

Retraction of the testicle is of rare occurrence. The digestive organs almost invariably suffer, and there is complete anorexia. The cachexia is not commonly highly developed. There is emaciation, and usually œdema of the lower extremities. General dropsy is rare; and it is a singular fact that the brain does not suffer, and the intellectual faculties often remain clear to the last.

Little can be done by treatment. The diet should be nutritious, unless there be hemorrhage. Astringents and iron are useful in warding off attacks of hematuria and the cachectic symptoms, especially general dropsy. Opium should be given to relieve the pain. Small local bleedings may be occasionally useful in the same way, and to relieve hemorrhage; but they should only be employed when milder means have failed. Counter-irritation can only prove injurious.

The diagnosis is sometimes exceedingly difficult, and mistakes have occasionally been made by even the most accurate observers. Our readers will find in Dr. Walshe's pages full directions for their guidance; by the accurate following of which the chances of error will be greatly diminished.

CANCER OF THE BLADDER.

Cancerous diseases of this viscus are much more frequent than is commonly supposed: 72 of the 8289 deaths, so often alluded to, were caused by it; and Dr. Walshe believes that if the term fungi, habitually applied to growths in that organ, were more correctly defined, the estimate would be much higher. Tuberiform encephaloid is the usual species, scirrhus in any form being comparatively rare. In most cases there is only one tumour; and the common seat is the triangular space between the orifices of the ureters and the urethra, and the parts immediately adjoining this space. It also occasionally springs from the fundus and anterior or posterior surfaces, but has very rarely been observed on the sides of the organ. The progress of the disease is the same as in other hollow viscera, but, as a general rule, ulceration is slow to occur. The bladder is generally contracted, but sometimes enlarged; its walls are usually hypertrophous, and the mucous membrane presents the columnar aspect. Evidences of cystitis are sometimes seen, and calculous disease frequently coexists. The prostate may or may not be similarly diseased, and the condition of the kidneys and upper part of the urinary passages is various; but it should be observed that the coexistence of primary cancer in the kidney with primary cancer in the bladder is, considering the intimate functional connexion of the parts, singularly rare. The primary disease is almost exclusively confined to the male sex, and does not appear to occur before the fortieth year.

Among the earliest symptoms is frequent and difficult micturition. Uneasiness, sometimes amounting to severe pain, and increasing after the urine is passed, is experienced at the neck of the bladder. In some cases there is complete retention, in others a full stream may be suddenly stopped. These variations are caused by the position and mobility or fixedness of the tumour. The urine is usually turbid, and often contains ropy matter, composed of pus modified by the evolution of ammonia from decomposed urea. Hemorrhage is frequent, often the first symptom, and occasionally the cause of death. In some cases tumour can be felt in the hypogastrium.

The diagnosis is most obscure, often impossible; but it is probable the microscope may afford assistance, by revealing the presence of cancer-cells in the urinary deposits.

The treatment should be merely palliative. To attempt the removal of the growths by operation would be to hasten the death of the patient.

### CANCER OF THE GENITAL ORGANS.

The extreme inequality which prevails in the proneness of the two sexes to cancerous disease of these parts, and of the breast, is well shown in the following table, constructed from the Paris registers:

| Of the Male. | | Of the Female. | |
|---|---|---|---|
| Testicle | 21 cases | Uterus | 2996 cases |
| Penis | 10 | Ovary | 64 |
| Scrotum | 7 | Vagina | 14 |
| Prostate | 5 | Vulva | 2 |
| | 43 | | 3076 |
| Breast | 5 | Breast | 1147 |
| | 48 | | 4223 |

*Male Organs.* Scirrhus is the species which most commonly invades the *penis.* A basis of this kind, however, not unfrequently gives origin to encephaloid vegetations. The disease may commence in almost all parts of the organ, but the glans and prepuce are by far its most common primary seat. It may originate either from a warty excrescence, or a pimple; or it may infiltrate the glans, or appear as a complication of venereal ulcerations. Phymosis, either congenital or acquired, is an exceedingly common accompaniment; and it appears probable that the irritation occasioned by this condition of the parts may act as an exciting cause of the disease in persons predisposed to cancer. Circumcision is, therefore, an advisable prophylactic measure, where the constitutional taint is known to exist. The duration of the disease is exceedingly various. It passes through the ordinary stages, and contaminates the lymphatic glands, the inguinal, according to M. Buret, becoming first affected if the superficial parts are involved, the pelvic, if the deeper seated. Amputation is the only possible remedy, and it affords but a very miserable chance of cure. M. Lisfranc has proposed to dissect off the prepuce when this is the diseased part, leaving the rest of the organ intact; but the measure is one of doubtful utility.

The *vesiculæ seminales* have been found cancerous in some cases, but nothing is known of the symptomatology or diagnosis of such a condition.

The disease is rare in the *prostate.* When it exists it is usually of the encephaloid species. All the lobes of the gland may be affected, but the middle is specially liable. There is nothing peculiar in the symptoms, and the treatment must be merely palliative.

It is in the *testicle* that we find the most numerous examples of carcinoma, and here, too, encephaloid is the common species, true scirrhus being very rare. The growth, when of large bulk, often has the characters of a composite tumour: and yellow discoloration, usually ascribed to the association of tuberculous matter, but most probably produced by softened fibrin, is very frequently observed. Both testicles are prone to the disease, but there is no example on record of their being, either simultaneously or consecutively, affected in the same individual. In the great majority of cases the body of the testis is the part first involved; and it seems probable that the disease commences by infiltration of the intervascular interstices of the gland. The progress is the same as in other parts, but ulceration of the investing scrotum is slow to occur. The tunica vaginalis becomes early diseased; the arteries of the cord are greatly enlarged, when the tumour has acquired considerable bulk; and the subcutaneous veins exhibit the usual dilatation. Contamination of the lymphatic system invariably occurs, the lumbar glands being most commonly affected.

The lungs, liver, kidneys, brain, meninges, bones, especially of the spine, the mesenteric glands, vena cava, and heart, are among the observed seats of secondary cancer, when the testis is its primary nidus; and perhaps the implication of the brain and spine is slightly more frequent under these than any other circumstances.

The disease may occur at all ages; and it is here that external violence has been most observed as an exciting cause. Horse exercise should, therefore, be avoided by persons of cancerous descent.

Increase of bulk and hardness of some particular point are the symptoms first noticed. These advance progressively, until the organ may have acquired a large size, and have changed its natural ovoid for a spheroidal shape. The diagnosis is sometimes extremely difficult. *Simple hypertrophy* is known by its commonly affecting both testes; *simple chronic induration,* which in many of its symptoms closely resembles cancer, by its yielding to mercurial treatment. *Syphilitic induration* is accompanied by dull pain, which is generally worse at night, but never lancinating: the organ, though sometimes increased, is not unfrequently diminished in bulk, and, as it were, wrinkled, its investments retain their natural character; the density of the gland increases, and its suppleness and elasticity are lost. The history and effects of treatment will also help to correct error. *Tuberculous disease* scarcely occurs except in phthisical subjects. In the ulcerated state mistakes can hardly be made; in the chronic unulcerated condition, the diagnosis will be aided by the smaller bulk of the part, its more distinctly nodular character, and its extremely slow progress. *Fibrous tumour* is excessively rare; it might be distinguished by its great weight, and the absence of pain and constitutional symptoms. *Cystoid and cysto-sarcomatous growths* present great difficulties; but they grow more slowly, are not productive of pain, and do not affect the cord, lymphatic glands, or constitution.

Compression, and the internal use of the iodide of arsenic, combined with the local inunction of the iodide of lead ointment, is a plan of treatment which deserves full trial. The results of extirpation are melancholy in the extreme.

*Female Organs.* We need not dwell upon cancer of the external genitals. The disease occurs in the *ovaries* either as a primary affection, as an extension or a sequence of the same morbid state in the uterus, or consecutively to the growth of cancer in distant and unconnected parts; but this is rare.

Scirrhus and encephaloid, both tuberiform and infiltrated, occur in this organ. The latter is the most common. Dr. Walshe has never met with colloid himself, nor found any satisfactory account of its being observed by others. The cancerous matter sometimes exists alone, but more commonly it is superadded to other morbid formations, and especially to multilocular cysts. Under such circumstances it is deposited in various forms on the walls of the cysts; but it is necessary to caution young observers against the frequent mistake of regarding as true scirrhous productions simple cysts the walls of which have been converted into fibrous tissue. The disease is usually confined to one ovary, though both are not unfrequently implicated. The morbid mass contracts adhesions with surrounding parts in many cases, and these adhesions have been found extensive where there had been no symptoms of peritoneal irritation—a fact of obvious practical importance. Of the special causes nothing is known, and the symptoms are merely those of ovarian enlargement in general. That it is no easy matter at all times to diagnosticate with accuracy the precise organ with which an abdominal tumour is connected, our readers are well aware; but we have not space to enter upon the question here. Let it then be granted that the growth, in any given case, is ovarian, how are we to determine its *nature?* If there be marked cancerous disease in any other organ, or if the cachexia be established, we have good ground for believing that the disease in question is of a similar kind. But should these helps be absent, we must be guided by the local characters of the tumour.

"*Fibrous tumours of the ovary* may, when large, be distinguished from scirrhus by their size; from encephaloid by their hardness, inferior elasticity, smoother non-lobulated surface, and uniform consistence in every part. The condition of the patient's health may facilitate the distinction. *Unilocular cysts* are of globular outline and non-lobulated; they usually contain a serous fluid, in which case the fluctuation of this will readily prove that the mass of the tumour does not consist of cancerous matter; and even where the contents are of a gelatinous character, the phenomenon is sufficiently well marked to prevent mistake. They are frequently moveable, and are of slow growth. The constitution suffers little in this disease. *Multilocular cysts,* on the contrary, either from the coexistence of their contents (firm, jelly-like matter, blood, fluid or coagulated, atheroma, acephalocysts, pilous and fatty substance), or from the non-communication of their cells, rarely give rise to more than very obscure fluctuation, which may almost be confounded with the elastic doughiness of encephaloid. Careful examination will, however, enable the observer to avoid this error.

"It is not enough, however, that we be able to affirm that the entire mass of the tumour is not composed of cancer—an extremely rare occurrence; it is necessary to ascertain whether, and to what extent, encysted growths contain carcinomatous formations in their walls. In certain cases this may be done by the detection, both by manual examination and percussion, of irregular indurated masses contrasting with the softer divisions of the remainder of the tumour; when the cysts are exceedingly tense from thorough repletion with fluid, the sign of lobulation may not be ascertainable: in this state of things evacuation of the fluid by paracentesis will remove this difficulty in the way of the diagnosis. Nevertheless the discovery of these lobulated masses is not a certain sign of cancerous formation; bunches of sub-cysts, with gelatinous or albuminous contents (I mean as respects their outward appearance), aggregated in the parietes of larger cysts, give rise to a precisely similar sensation. When hydatid fremitus exists in a marked manner, the presence of *acephalocysts* may be safely announced. Rapidity of course of the disease furnishes one of the best evidences of encephaloid complication."

In regard to treatment, Dr. Walshe strongly urges the full trial of iodine, both externally and internally, in the way before noticed. His own experience enables him to affirm that the progress of the disease may be thus arrested, and the size of the tumour in some cases actually diminished. In respect of puncture, it should be remembered that when there is encephaloid matter in the cysts, the evacuation of their fluid contents often occasions a more rapid deposition; the operation should, therefore, be undertaken only under the pressure of necessity.

We should now proceed to the consideration of the very important subject of *uterine cancer,* which is most ably treated by our author; but we have already wellnigh exhausted our assigned limits, and we deem it more advisable to employ what little space yet remains in bringing before our readers some points in the history of the disease which are less generally known; and we, therefore, purpose to pass entirely by this section and the next succeeding, which treats of *cancer of the breast,* with the simple remark, that in them will be found lucid descriptions of the morbid conditions, clear rules for diagnosis, and judicious advice in regard to treatment.

CANCER OF THE ORGANS OF INNERVATION AND APPENDAGES.

*Encephalon.* Every part of the encephalon has been the observed seat of cancer; but it is by far the most common in the cerebral hemispheres. Each of the three species is found here, but encephaloid much the most frequently. It exists in both the tuberiform and the infiltrated forms, the size and extent varying much. Generally, the brain contains but one cancerous growth. In some instances the tumours appear actually continuous with the surrounding brain; in others they are surrounded by a pseudo-cyst; while, most commonly, without such an envelope, they seem deficient in intimate connexion with the tissues around. The disease may be either primary or secondary. The state of the non-cancerous portions of the brain varies: it may be quite healthy; or exhibit punctiform injection, and flattening of the convolutions; or the neighbouring parts may be in a state of colourless softening. It is a singular fact that subarachnoid and ventricular effusion is usually very inconsiderable, and often altogether wanting.

The symptoms are unhappily obscure. Pain usually exists; but its characters, severity, and persistence vary within wide limits, and it is sometimes absent throughout. It is occasionally limited to a single spot, but is more frequently without defined position. The sensibility of the limbs and trunk may be diminished, increased, or perverted. Paralysis may or may not be present; it is sometimes hemiplegic, sometimes paraplegic, or it may be universal. It usually comes on gradually, but it may be developed at once. Partial or general convulsions are not uncommon. The intellect may or may not be affected. There is usually some derangement of vision, and deafness has been noted in some cases; but the senses of smell and taste rarely suffer. The nutritive functions remain long unimpaired, but eventually they fail, and the patient becomes emaciated. The skin rarely acquires the yellow tint.

Colourless softening is the affection most likely to be confounded with cancer. The marks of distinction, as stated by M. Durand-Fardel, are the less frequency of cephalalgia, its being rarely limited to one side of the head, and almost always frontal; the absence of complete amaurosis, and the extreme rarity of convulsions in non-paralysed parts.

But even when the evidence of a tumour in the brain is decisive, it may not be cancerous; for various other productions are found in the same situation. Experience has not yet given any sure marks of diagnosis; but it should be remembered, with reference to one form, the tubercular, that M. Louis has proved that, with the rarest exceptions, the presence of tubercle in any organ, involves, in subjects aged upwards of fifteen, its existence in the lungs. Careful examination of these organs will, therefore, render assistance.

The treatment can only be palliative; it should be directed to the removal of irritation in the brain.

When the *cerebellum* is the seat of disease, the pain is in some cases refeired to the occiput and nucha, but this is by no means invariable. The intellect is rarely affected. In a very few cases only has there been defective co-ordination of muscular action, which ought to have been frequent had the doctrines of certain physiologists been founded on truth. Hemiplegia is excessively rare; paraplegia still more so. Convulsions are more common. Complete or incomplete amaurosis has been often observed. Deafness has never occurred, unless the portio mollis has been actually implicated; and the phrenological views of the functions of this organ receive no support from recorded cases of this disease.

CEREBRAL MENINGES AND CRANIUM.

Under this head Dr. Walshe includes those cases of intracranial cancerous disease, which are practically distinguished by the morbid production perforating the cranium, and forming a fungous growth on its external surface; as likewise those where it originates in the meninges, without making its way outwards through the skull; and those excessively rare cases, wherein the disease is alleged to have formed in the subpericranial cellular membrane. The whole chapter is one of extreme interest and value; but a mere glance must suffice us here.

The disease, which is usually encephaloid, but in some few instances true scirrhus, may originate in any one of the following situations: 1, the external or the internal lamina of the dura mater; 2, pia mater; 3, cerebral substance; 4, cranial bones, generally the diploe, but occasionally the compact tissue; 5, subpericranial cellular tissue. In most cases there is only one tumour, but sometimes they are very numerous. They have been found at all ages, and with nearly equal frequency in both sexes. The symptoms vary according as the tumour continues still within the cranium, or has made its way externally. Under the first-mentioned circumstances they are the ordinary evidences of compression, sometimes developed suddenly, but most commonly by slow degrees. When the bony covering has been perforated, which is effected either by carcinomatous opening of the osseous tissue, or, as is more usual, by atrophous absorption, the cancerous formation protrudes through the adventitious foramen in the form of a fungous tumour. This tumour may possess one or more of several characters, which, if combined in any number, are pathognomonic. It has the lobular surface and soft elasticity of encephaloid; it is immovable, yet non-adherent to the skin, which retains its natural appearance. The irregular edges of the perforation may sometimes be traced round it. It is the seat of two pulsations—one synchronous with the arterial pulse, the other corresponding with the rise and fall of the brain during respiration. It may be reduced either wholly or in part, and thus give rise to symptoms of compression; when left to itself, it recovers its previous appearance and size. It is not usually tender to the touch, but is often the seat of darting pain, which may be relieved by *gentle* pressure. Death usually occurs before ulceration has taken place.

But almost all these symptoms may be absent, and this constitutes the great difficulty in diagnosis. The disease may also be confounded with other maladies, as *encephalocele;* which, however, unless the result of accident, is almost peculiar to new-born infants—is, with the rarest exceptions, seated on the sutures or fontanelles, where cancer is not observed, is of equable consistence throughout, and passes through an opening with smooth and even edges. *Erectile tumours,* when they have attained any size, usually affect the condition of the skin, and their pulsations are more influenced by the general state of the circulation, and can be more readily controlled by pressure on the carotid. *Aneurism* will be known by the blowing murmur and the absence of opening in the skull. *Encysted tumours* and *abscess* could hardly be mistaken, unless the observer were very careless. *Cephalhæmatoma* exists in new-born infants alone; and only two cases of perforating fungus at this age are on record.

As respects treatment, Dr. Walshe recommends the trial of gentle compression. The lamentable results of surgical interference are strikingly shown in a table at p. 520.

Here we must pause. Our notice has extended to a much greater length than we had anticipated, and yet we feel that we have done our author but scant justice; and now we leave altogether untouched the very interesting and valuable information which he has collected regarding the cancerous affections of the spinal column and its contents, the nerves, skin, bones, and subcutaneous cellular tissue. To these, as well as all other portions of the volume, we again earnestly direct the attention of our readers; being confident that nowhere will they find a safer guide, or a more intelligent teacher than Dr. Walshe.

---

